# The Ventral Striatum is a Key Node for Functional Recovery of Finger Dexterity After Spinal Cord Injury in Monkeys

**DOI:** 10.1093/cercor/bhz307

**Published:** 2019-12-09

**Authors:** Michiaki Suzuki, Kayo Onoe, Masahiro Sawada, Nobuaki Takahashi, Noriyuki Higo, Yumi Murata, Hideo Tsukada, Tadashi Isa, Hirotaka Onoe, Yukio Nishimura

**Affiliations:** 1 Neural Prosthesis Project, Dementia and Higher Brain Function, Tokyo Metropolitan Institute of Medical Science, Setagaya, Tokyo 156-8506, Japan; 2 Department of Developmental Physiology, National Institute for Physiological Sciences, Okazaki, Aichi 444-8585, Japan; 3 Department of Physiological Sciences, School of Life Science, SOKENDAI (The Graduate University for Advanced Studies), Hayama, Kanagawa 240-0193, Japan; 4 Department of Neuroscience, Graduate School of Medicine and Faculty of Medicine, Kyoto University, Sakyo, Kyoto 606-8501, Japan; 5 Japan Society for the Promotion of Science, Chiyoda, Tokyo 102-0083, Japan; 6 Laboratory for Pathophysiological and Health Science, RIKEN Center for Biosystems Dynamics Research, Kobe, Hyogo 650-0047, Japan; 7 Department of Neurosurgery, Graduate School of Medicine, Kyoto University, Sakyo, Kyoto 606-8501, Japan; 8 Human Informatics Research Institute, National Institute of Advanced Industrial Science and Technology, Tsukuba, Ibaraki 305-8568, Japan; 9 Central Research Laboratory, Hamamatsu Photonics, Hamamatsu, Shizuoka 434-8601, Japan; 10 Intitute for the Advanced Study of Human Biology (WPI-ASHBi), Kyoto University, Sakyo, Kyoto 606-8501, Japan; 11 Human Brain Research Center, Graduate School of Medicine and Faculty of Medicine, Kyoto University, Sakyo, Kyoto 606-8507, Japan

**Keywords:** finger dexterity, functional recovery, nonhuman primate, plasticity, spinal cord injury

## Abstract

In a recent study, we demonstrated that the ventral striatum (VSt) controls finger movements directly during the early recovery stage after spinal cord injury (SCI), implying that the VSt may be a part of neural substrates responsible for the recovery of dexterous finger movements. The VSt is accepted widely as a key node for motivation, but is not thought to be involved in the direct control of limb movements. Therefore, whether a causal relationship exists between the VSt and motor recovery after SCI is unknown, and the role of the VSt in the recovery of dexterous finger movements orfinger movements in general after SCI remains unclear. In the present study, functional brain imaging in a macaque model of SCI revealed a strengthened functional connectivity between motor-related areas and the VSt during the recovery process for precision grip, but not whole finger grip after SCI. Furthermore, permanent lesion of the VSt impeded the recoveryof precision grip, but not coarse grip. Thus, the VSt was needed specifically for functional recovery of dexterous finger movements. These results suggest that the VSt is the key node of the cortical reorganization required for functional recovery of finger dexterity.

## Introduction

Neuronal mechanisms underpinning functional recovery after neuronal damage, such as spinal cord injury (SCI) or stroke, have been investigated widely in humans and animal models. Dexterous finger movements, such as precision grip, depend largely on the corticospinal tract (CST) in higher primates and are severely impaired immediately after neuronal damage (Lawrence and Kuypers 1968a, 1968b; Nishimura et al. 2007a, 2007b, 2009, 2011; Higo et al. 2009, 2018; Sugiyama et al. 2013; Sawada et al. 2015). Following a CST lesion at the midcervical segment in nonhuman primates, dynamic plastic changes in the primary motor cortex (M1), premotor cortex (PM), and spinal circuits are associated with recovery of dexterous finger movements (Sasaki et al. 2004; Nishimura et al. 2007a; Higo et al. 2009, 2018; Tohyama et al. 2017; Chao et al. 2018). However, the contribution of structures up-stream of the motor cortices to recovery after SCI remains unclear. Recently, we demonstrated that the ventral striatum (VSt), which is largely known as a key subcortical node for processing motivation and reward (Schultz et al. 1992; Aberman and Salamone 1999; Pessiglione et al. 2007; Bichot et al. 2011; Schmidt et al. 2012), facilitates activity of the M1 and is directly involved in the control of finger movements in the early recovery stage after SCI (Sawada et al. 2015). Although the VSt is not thought to be involved directly in motor control, this finding implies that the VSt may be an essential node for functional recovery following SCI. However, it is unclear whether there is a causal relationship of the VSt to motor recovery, and whether the VSt shows specificity for the recovery of dexterous finger movements or finger movements in general after SCI.

In the present study, to investigate the functional significance and causal contribution of the VSt to functional recovery of dexterous finger movements after SCI, we performed functional brain imaging using positron emission tomography (PET) and bilateral VSt lesion studies. Our results demonstrate that neuroplastic functional reorganization of the VSt-motor networks occurs and the VSt causally contributes to functional recovery of dexterous finger movements after SCI.

## Materials and Methods

Nine adult macaque monkeys (3.5–8.1 kg) were used in the present study (see details about subjects’ information in Supplementary Table 1). Three monkeys [*Macaca fuscata*: Monkey H (female); *Macaca mulatta*: Monkeys K (male) and TF (male)] were used for the PET experiments. Behavioral data regarding performance of precision grip in the slit task (see details in Behavioral test for assessment of dexterous finger movements section) and some of the PET data concerning the precision grip and the control tasks, except for the whole finger grip task (see details in Behavioral tasks in the PET scanner section), have been published in previous articles (Supplementary Table 2) (Nishimura et al. 2007a, 2007b, 2011). For the present study, relevant PET data were reanalyzed using improved statistical methods described below with a different focus. The previous study investigated the neural substrate involved only in precision grip task, while the present study focused on the significance of the difference of neural substrate underlying functional recovery between the precision and whole finger grip. Six monkeys [*Macaca mulatta*: Monkeys Ju (female) and Na (female); *Macaca fuscata*: Monkeys Sh (female), M (male), T (female), and R (female)] were used for the VSt intervention experiments. Behavioral data concerning the performance of precision grip in the slit task in Monkey M, T, and R were partly obtained from our previous study (Supplementary Table 3) (Sawada et al. 2015). Throughout the experiments, the monkeys were housed in individual cages that were temperature- and light-controlled (23–26 °C and 12-h on/off cycle, respectively). They were fed regularly with diet pellets and had free access to water. They were monitored closely and animal welfare was assessed on a daily basis or, if necessary, several times a day. All procedures involving animals were approved by the Committee for Animal Experimentation of the National Institutes of Natural Sciences (Approval No. 14A126), the Central Research Laboratory in Hamamatsu Photonics (Approval No. HPK-17-02), and the Amami Wild Animal Research Center Inc. (Approval No. H17001). All procedures involving animals were performed in accordance with National Institutes of Health Guideline for the Care and Use of Laboratory Animals.

### Behavioral Test for Assessment of Dexterous Finger Movements

**Figure 1:**
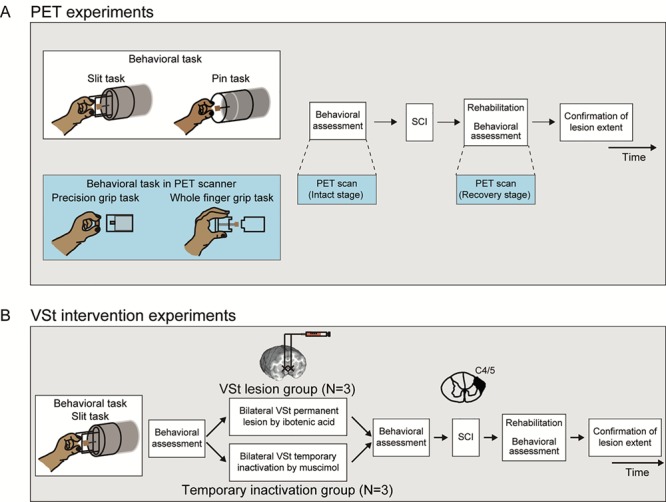
Experimental procedure of the PET experiments and the VSt intervention experiments. (*A*) Experimental procedure of the PET experiments. All three monkeys (Monkeys H, K, and TF) were trained and tested in the slit task and the pin task before and after SCI. In addition, the monkeys were also trained in the precision grip task, the whole finger grip task, and the control task in the PET scanner (see Materials and Methods and Supplementary Fig. 1). At the end of the experiment, the lesion extent of the spinal cord was confirmed by histological analyses. (*B*) Experimental procedure of the VSt intervention experiments. All monkeys in the VSt lesion group and the temporary inactivation group were trained and tested in the slit task before and after the bilateral VSt lesion or temporary inactivation and SCI, respectively. At the end of the experiment, the extent of the VSt lesions and the spinal cord lesions was confirmed by histological analyses.

To assess finger dexterity, all nine monkeys were trained to reach-grasp-retrieve a small morsel of sweet potato (about 5 × 5 × 5 mm) through a narrow vertical slit, which guides the monkeys to use a precision grip (Slit task; Fig. 1*A*,*B*). Three monkeys used in the PET experiments were trained on an additional reach-grasp-retrieve task, in which a morsel was attached to a pin inserted through the bottom of a horizontal tube positioned in the midsagittal plane (Pin task; Fig. 1*A*). During training/rehabilitation and test sessions, the contralateral hand to the task engaged hand was constrained. Each training session consisted of ~100 trials, while each testing session consisted of 30 trials. A digital video camera (33 frames/s) was used to record the reach-retrieval sequences from a lateral view. After SCI, we observed two kinds of grasping strategies, a precision grip and a coarse grip, during the course of recovery. A successful precision grip was defined as grasping and retrieving a morsel of sweet potato with the pads of the index finger and thumb (index finger-to-thumb opposition) without dropping it. A coarse grip was defined as when the monkeys retrieved a morsel using other gripping strategies, such as whole-finger grip by a clenched hand, holding a morsel between the pad and nail of the fingers, and raking a morsel with the index finger, also without dropping it. The rate (%) of precision grip and that of coarse grip were calculated as the number of successful trials with either grip divided by 30 trials, respectively.

### Treatments Related to Surgeries

All surgeries described below were performed under sterile conditions and general anesthesia, starting with a combination of intramuscular injections (i.m.) of ketamine (10 mg/kg) and xylazine (1 mg/kg), followed by intubation and isoflurane (1–1.5%) inhalation to maintain anesthesia throughout surgery. Ampicillin (40 mg/kg) was injected as an antibiotic before surgery, and dexamethasone (0.825 mg/kg) and ketoprofen (0.4 mg/kg) were administered to reduce postoperative pain and inflammation after surgery.

### SCI

Lateral CST (l-CST) lesions were performed, as described in detail elsewhere (Sasaki et al. 2004; Nishimura et al. 2007a, 2009; Higo et al. 2009, 2018; Sawada et al. 2015). Under anesthesia, the border between the C4 and C5 segments was exposed by laminectomy of the C3 and C4 vertebrae, and a transverse opening was made in the dura. The dorsal part of the lateral funiculus was transected from the dorsal root entry zone ventrally to the level of the horizontal strip lesion. The lesion was extended ventrally at the most lateral part of the lateral funiculus. The skin and back muscle incisions were closed with nylon or silk sutures.

### Subjects for the PET Experiments

Three monkeys (Monkeys H, K, and TF) were used for the PET experiments. As described above, all three monkeys were trained intensively to perform the pin task and the slit task in the monkey chair. Monkey H performed the task with the right hand and received the l-CST lesion on the right side. Monkeys K and TF performed with the left hand and received the l-CST lesion on left side. Both the behavioral test and training sessions were conducted before and after SCI (Fig. 1*A*). After taking an anatomical image of the brain using MRI, an acrylic head holder was attached to the skull and used for fixation of the monkey’s head on the monkey chair during the PET scanning.

### Behavioral Tasks in the PET Scanner

The monkeys were trained to sit in the monkey chair and to perform the precision grip task (Fig. 1*A*) at constant intertrial interval (once every 5 s) with the hand affected by SCI in both the training room and the PET scanner. To compare the neural substrates between highly fractionated finger movement and gross finger movement, the monkeys were also trained to perform an additional whole finger grip task as gross-finger movement, in which they were required to reach-grasp-retrieve a piece of sweet potato from an acrylic cylinder by whole-finger grasping (Fig. 1*A*). The morsel of sweet potato was attached to the rear side of the cylinder. In both tasks, after successful grasping, the monkey ate the sweet potato. Last, the monkeys were trained on a control task (Supplementary Fig. 1), in which the morsel of sweet potato was placed on the tip of a rod attached to a long tube that was presented directly to their mouth while both arms were restricted (Nishimura et al. 2007a, 2007b). The food piece approached the monkeys from the same position and angle as in the precision grip/whole finger grip task. The pace of food delivery in this control procedure was the same as that used in the precision grip/whole finger grip task.

### PET Scans

**Figure 2:**
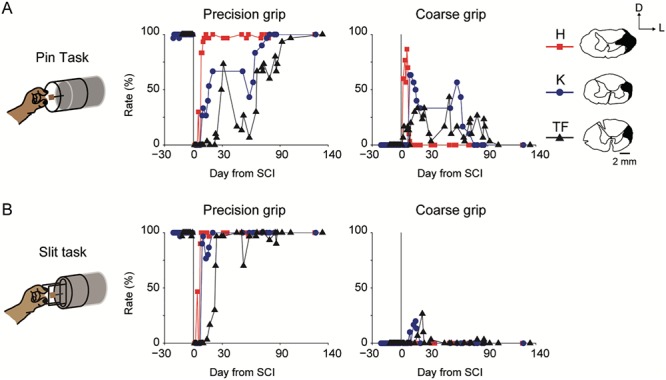
Recovery time course of finger movements after SCI in the PET experiments. (*A*) Rate of the precision grip and coarse grip in the pin task (*N* = 3; Monkeys H, K, and TF). Illustrations of coronal spinal cord sections indicating lesion extent (black hatch) of the l-CST at the C4/C5 segment in individual monkeys. Illustrations of Monkey H are flipped left and right because of the lesion side being opposite to that of the other monkeys. Those illustrations were reprinted with permission from AAAS (Nishimura et al. 2007a). D, dorsal; L, lateral. The rate (%) of precision grip and that of coarse grip were calculated as the number of successful trials with either grip divided by 30 trials, respectively (see Materials and Methods). (*B*) Rate of the precision grip and coarse grip in the slit task (*N* = 3; Monkeys H, K, and TF).

PET scans were performed with an SHR7700 camera (Hamamatsu Photonics K.K. Japan), which has 31 slices with a center-to-center distance of 3.6 mm and its axial and transaxial resolution is 2.6 mm at full-width-half-maximum (FWHM) (Watanabe et al. 1997). A series of PET scans measuring the regional cerebral blood flow (rCBF) as an index of the neuronal activity, was conducted during the intact and recovery stages, as described in our previous studies (Nishimura et al. 2007a, 2011). PET scans after SCI were conducted when the success rate for precision grip in the slit task reached 80–100% (precision grip in Fig. 2*B*). In Monkey H, PET scans after SCI were conducted between postoperative Days 15 and 112. In Monkey K, PET scans after SCI were conducted between postoperative Days 22 and 115. In monkey T, PET scans after SCI were conducted between postoperative Days 36 and 122. Twenty-four scans were conducted on Monkeys H and K for all the three tasks during the intact stage and 48 scans during the recovery stage. Twenty-seven scans were conducted in Monkey TF during the intact stage and 55 scans during the recovery stage. Each monkey was allowed to start the behavioral task (20 trials) at approximately 10 s before the start of the PET scan with the delivery of a bolus of [^15^O]H_2_O via a cannula preinserted into the sural vein. The sessions of the precision grip task, the whole finger grip task, and the control task were tested in a random order. PET data were collected for 80 s (one 40 s frame followed by four 10 s frames).

### Statistical Analysis of PET Data

Reconstructed brain images (voxel size, 1.2 × 1.2 × 3.6 mm) were scalped and smoothed with a 4.0 mm FWHM isotropic kernel, and were processed using statistical analyses available in the current parametric mapping (SPM8) software. PET images obtained from the scan sessions were summated for their first 60 s epochs, and were used for statistical analysis. The significant foci of intersubject data were assessed using the analysis of covariance with global normalization. For the intersubject analysis, brain shapes of individual [^15^O]H_2_O-PET images were normalized morphologically to a pseudobrain template [^18^F]FDG-PET image (voxel size, 0.5 × 0.5 × 0.5 mm), which was preadjusted to the standard MRI template. In order to identify the specific activity that reflects the main effect of functional recovery, we defined the contrast as [precision grip (whole finger grip) task at the recovery stage—control task at the recovery stage]—[precision grip (whole finger grip) task at the intact stage—control task at the intact stage]. The statistical threshold was set at *P* < 0.01, uncorrected for multiple comparisons (*t* > 2.33). To determine the anatomical localization of activated foci, the SPM{t} PET images were coregistered precisely to the template MRI. For functional connectivity analysis, voxel of interest (VOI) for the M1 and VSt on the contralesional hemisphere (co-M1, co-VSt) were determined by the results of the main effect of the functional recovery of precision grip. VOI for co-M1 was the entire region of significant functional activation thresholded at *P* < 0.001 (*t* > 3.1) consisting of 720 voxels, while that for co-VSt was the entire region of significant functional activation thresholded at *P* < 0.01 (*t* > 2.3) consisting of 190 voxels. The regions where rCBF values were correlated with those of the co-M1 or co-VSt during the precision grip task or the whole finger grip task were also determined by interregional correlation analysis using SPM. The statistical threshold was set at *P* < 0.01, uncorrected for multiple comparisons (*t* > 2.38 for the intact stage, *t* > 2.35 for the recovery stage).

### Subjects for the VSt Intervention Experiments

Six monkeys were used for the VSt intervention experiments. Monkeys Ju, Na, and Sh received the permanent bilateral VSt lesion (VSt lesion group). Monkeys M, T, and R received the temporary inactivation of the bilateral VSt (temporary inactivation group). As described above, all six monkeys were trained intensively to perform the slit task in their home cage (VSt lesion group) or in the monkey chair (temporary inactivation group). The monkeys performed with the left hand and received the l-CST lesion on the left side. Behavioral training and test sessions were conducted before and after the bilateral VSt lesion or temporary inactivation and before and after SCI (Fig. 1*B*).

### Bilateral VSt Intervention

To define the location of the VSt, we performed a craniotomy and identified the rostral arcuate sulcus spur in each hemisphere as a reference surface position. Ibotenic acid [15 μg/μL, dissolved in 0.1 M phosphate-buffered saline (PBS), pH 7.4] was injected to destroy neurons in the VSt, and the injection site was determined by stereotaxic coordinates according to the atlas (Paxinos et al. 2009). Multiple injections by a 10-μL Hamilton syringe were performed to lesion the entire VSt, with five tracks into each hemisphere separated from each other by 2 mm. In each track, injections were made at one or two sites separated by 2 mm in depth. Ibotenic acid was injected at a rate of 0.2 μL/min (nine sites/hemisphere, 1 μL/site). In order to compare the effect of temporary inactivation of the VSt on functional recovery after SCI with that of a permanent lesion, a temporary inactivation of the VSt was performed by injecting muscimol, a γ-aminobutyric acid type A (GABA_A_) receptor agonist [5 μg/μL, dissolved in 0.1 M phosphate buffer (PB), pH 7.4], into the bilateral VSt (eight sites/hemisphere, 1 μL/site) in monkeys of the temporary inactivation group before SCI, as described previously (Sawada et al. 2015). From our previous experiences (Nishimura et al. 2007a; Tsuboi et al. 2010; Murata et al. 2015; Sawada et al. 2015), the effect of muscimol disappears completely in 2 days after injection, indicating that muscimol induces a temporary effect.

### Histological Confirmation of the Lesion Extent

At the end of all these experiments, the monkeys were deeply anesthetized with an overdose of sodium pentobarbital (50–75 mg/kg, i.v.) and perfused transcardially with 0.1 M PBS (pH 7.4), followed by 4% paraformaldehyde in 0.1 M PB (pH 7.4). The spinal cord and brain were removed immediately and immersed successively in 10%, 20%, and 30% sucrose in 0.1 M PB (pH 7.3). The specimens were cut serially into coronal sections of 50 μm thickness on a freezing microtome. All sections were processed for Nissl-staining with 1% cresyl violet. We used photomicrographs of the spinal cord lesion and the VSt lesion to determine the lesion extent. The extent of the VSt lesion in three monkeys (VSt lesion group) was defined by the area of gliosis.

## Results

### VSt-M1 Linkage

Three macaque monkeys (Monkeys H, K, and TF) were assigned to the PET experiments. To assess the recovery course of finger dexterity, all three monkeys were trained in the pin task and the slit task (Fig. 1*A*, see Materials and Methods) before SCI. Retrieving a morsel with the pads of the index finger and thumb without dropping it in both tasks was categorized as a precision grip, while retrieving a morsel using other gripping strategies without dropping it was categorized as a coarse grip (see details in Materials and Methods). After sufficient training (>2 weeks), in which the monkeys showed a stable success rate of precision grip, the monkeys received the l-CST lesion at the border between the C4 and C5 segments. Finger movements were impaired immediately after the SCI (Fig. 2*A*,*B*). The monkeys began rehabilitative training of food retrieval with the affected hand on the day following the SCI. Immediately after the SCI, the monkeys tried to retrieve the morsel from the tip of a pin in the pin task by coarse grip such as whole-finger grip by a clenched hand, but this behavior disappeared at approximately 3 months after the SCI (Fig. 2*A*). At approximately 10 days after the SCI, the monkeys began to perform precision grip, which was fully recovered at approximately 3 months after the SCI. Thus, the recovery process was characterized by the grasping strategy that changed gradually from a coarse grip to a precision grip depending upon the recovery of finger dexterity. In the slit task, which involved retrieving the target through a narrow vertical slit, full recovery of precision grip was achieved within 1 month after SCI (Fig. 2*B*). On the basis of the time course of recovery in the slit task, we performed PET scanning during the intact stage and the recovery stage (see Materials and Methods).

**Figure 3:**
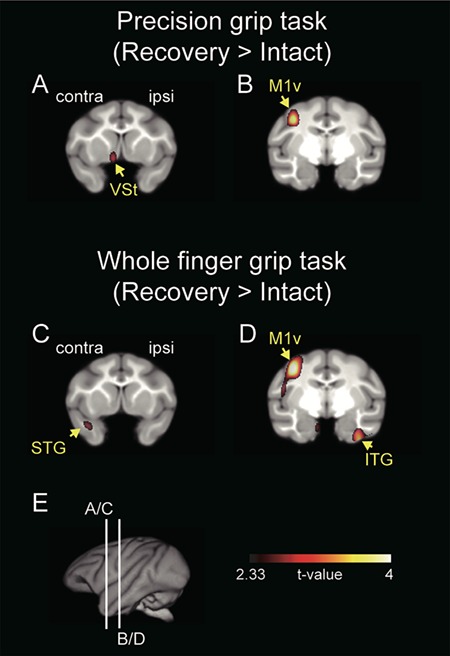
Increased brain activation related to functional recovery during the precision grip task and whole finger grip task. (*A–D*) Results show averaged data from the three monkeys (Monkeys H, K, and TF). Brain areas with significantly increased rCBF (*P* < 0.01, uncorrected for multiple comparisons) are superimposed on a template brain MRI of macaque monkeys. Other brain areas showing significant increased activation are presented in Supplementary Figure 2 and Supplementary Table 4. Coronal sections including the VSt are shown in (*A*) and (*C*). Coronal sections including the M1 are shown in (*B*) and (*D*). The significance level is in terms of *t*-values represented on a color scale. (*E*) Lines (A/C) and (B/D) indicate the levels of coronal sections A/C and B/D, respectively. Contra, contralesional; ipsi, ipsilesional; M1v, ventral aspect of the primary motor cortex; STG, superior temporal gyrus; ITG, inferior temporal gyrus.

To clarify the specific involvement of the VSt in the neural substrate for functional recovery of dexterous finger movements, we examined rCBF using PET during the precision grip or the whole finger grip task and analyzed the difference in brain activation between the tasks before and after SCI. All of the three monkeys were fully trained (>1 month) in the precision grip task and whole finger grip task in the PET scanner before SCI, as described in Materials and Methods (Fig. 1*A*). After SCI, recovery of both precision and whole finger grips was associated with increased activity in the ventral aspect of the contralesional M1 (co-M1v, *P* < 0.01, uncorrected; Fig. 3*B*,*D*), while increased activity in the contralesional VSt (co-VSt) was associated only with recovery of precision grip (*P* < 0.01, uncorrected; Fig. 3*A*). The location of increased activity in the co-M1v after SCI was similar to the location of the precision grip task-related activity in monkeys without SCI (Nishimura et al. 2007a, 2007b). Other sensorimotor-related areas, such as the contralesional primary somatosensory cortex (S1), also showed increased post-SCI activation relative to pre-SCI activation in both tasks (*P* < 0.01, uncorrected; Supplementary Fig. 2 and Supplementary Table 4).

**Figure 4:**
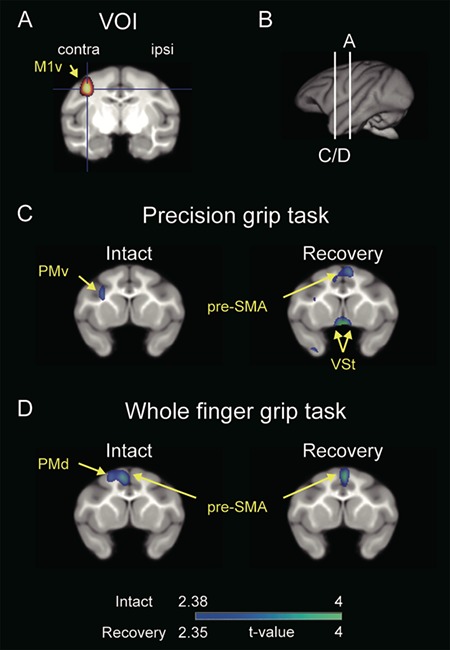
Functional connectivity between the contralesional M1 and VSt during recovery. (*A*) VOI for the ventral aspect of the contralesional M1 (co-M1v) was determined by the results of the main effect of the functional recovery shown in Figure 3*B*. VOI of the co-M1v was the entire region of significant functional activation thresholded at *P* < 0.001 (*t* > 3.1) consisting of 720 voxels. (*B*) Lines indicate the levels of coronal sections in A and C/D. (*C* and *D*) Coronal sections including the VSt are presented. Brain areas having a significant positive correlation with the co-M1v during (*C*) precision grip task and during (*D*) whole finger grip task are indicated on a template brain MRI by a color scale (*P* < 0.01, uncorrected for multiple comparisons) in the intact stage and the recovery stage. Results show averaged data from the three monkeys. See Supplementary Figure 3 and Supplementary Table 5 for other brain areas showing significant positive correlation with the co-M1. contra, contralesional; ipsi, ipsilesional; M1v, ventral aspect of the primary motor cortex; PMv, ventral premotor area; PMd, dorsal premotor area.

Furthermore, to clarify the functional linkage between the co-M1v and the co-VSt underlying functional recovery, we performed voxel-based correlation analyses before and after SCI. VOI for the co-M1v (Fig. 4*A* and Supplementary Fig. 3*A*) was determined by the results of the main effect for the functional recovery of precision grip (Fig. 3*B*). During this precision grip task, a significant positive correlation between co-M1v and the bilateral VSt was observed during the recovery stage (*P* < 0.01, uncorrected; recovery in Fig. 4*C*), but not during the intact stage (intact in Fig. 4*C*). In contrast, for the whole finger grip task both before and after SCI, no-significant correlation between the co-M1v and the VSt was observed (Fig. 4*D*). Thus, VSt-M1 linkage was associated with functional recovery of precision grip, but not whole finger grip.

### Large-Scaled Connectivity Between VSt and Motor Networks

To further understand the large-scaled reorganization associated with functional recovery from SCI, we performed correlation analyses on regions throughout the brain. Summaries of multiple comparisons of the whole brain regions with the co-M1 and the co-VSt are shown in Supplementary Figures 3 and 4 and in Supplementary Tables 5 and 6. During the recovery of precision grip, significant positive correlations with the co-M1v were observed in sensorimotor areas, including the bilateral presupplementary motor area (pre-SMA), contralesional ventral premotor area (co-PMv), bilateral M1v, bilateral S1, bilateral putamen (Pu), contralesional thalamus (co-Th), and limbic areas, including the contralesional orbitofrontal cortex (co-OFC) and the bilateral VSt, as mentioned above (*P* < 0.01, uncorrected). These correlations were not observed preoperatively, but emerged only during the behavioral recovery (Supplementary Fig. 3*C* and *D*).

**Figure 5:**
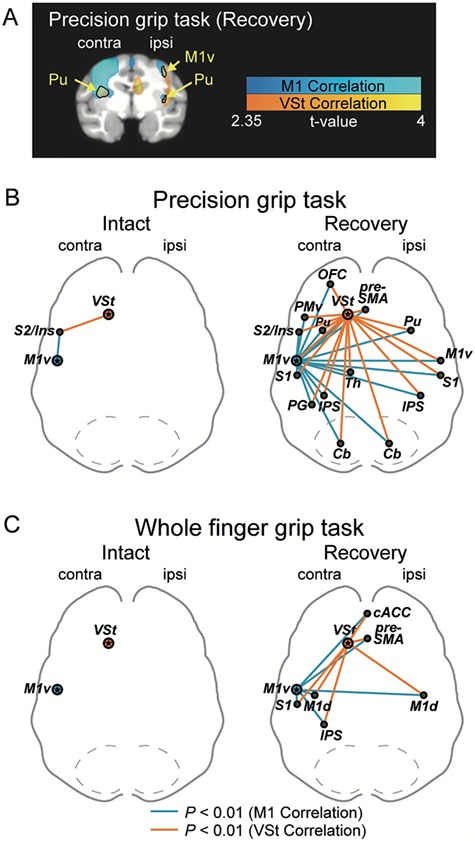
Overlapping functional connectivity between the VSt network and M1 network. (*A*) Example of overlapping functional connectivity. Brain regions with functional connectivity with the co-VSt (orange color, *P* < 0.01, uncorrected for multiple comparisons) and co-M1v (blue color, *P* < 0.01, uncorrected for multiple comparisons) are superimposed on the same template MRI. Overlapped areas are indicated by black outlines. Other brain areas are presented in Supplementary Figure 5. Results of the correlation analysis between the co-VSt and whole-brain are shown in Supplementary Figure 4 and Supplementary Table 6. (*B* and *C*) The functional connectivity with both co-VSt and co-M1v during (*B*) precision grip task and (*C*) whole finger grip task before and after SCI. Orange lines indicate significant functional connectivity with the co-VSt. Blue lines indicate significant functional connectivity with the co-M1v. Note that only brain areas having significant connectivity with both the co-VSt and the co-M1v are shown in these schemes (see Supplementary Fig. 3 and Supplementary Table 5 for results of correlation analysis with the co-M1 and Supplementary Fig. 4 and Supplementary Table 6 for results of correlation analysis with the co-VSt). contra, contralesional; ipsi, ipsilesional; OFC, orbitofrontal cortex; cACC, caudal anterior cingulate cortex; PMv, ventral premotor area; M1v, ventral aspect of the primary motor cortex; M1d, dorsal aspect of the primary motor cortex; S1, primary somatosensory cortex; S2, the secondary somatosensory cortex; Ins, insular cortex; PG, area PG in the posterior parietal cortex; Th, thalamus.

Interestingly, brain regions that showed positive correlations with the co-M1v, also showed positive correlations with the co-VSt in the recovery period (Supplementary Fig. 4). Figure 5*A* shows brain regions demonstrating significant positive correlation with the co-M1v (blue color) or the co-VSt (orange color), as well as brain regions that showed significantly overlapping connectivity with both the co-M1v and co-VSt (black outlines). In the intact stage during the precision grip task, overlapping connectivity was observed only with the secondary somatosensory cortex (S2)/insular region (intact in Fig. 5*B* and Supplementary Fig. 5*A*). However, during the recovery stage, the number of brain regions showing significant overlapping connectivity with both the co-M1v and co-VSt increased substantially. These brain regions include the sensorimotor network comprising the bilateral pre-SMA, co-PMv, bilateral M1v, bilateral S1, bilateral intraparietal sulcus (IPS) regions, contralesional area PG, subcortical areas [bilateral Pu, co-Th and bilateral cerebellum (Cb)], and limbic areas (including co-OFC) (recovery in Fig. 5*B* and Supplementary Fig. 5*B*). During performance of the whole finger grip task, no brain region showed overlapping connectivity in the intact stage (intact in Fig. 5*C* and Supplementary Fig. 5*C*). However, during the recovery period, reliable overlapping connectivity with bilateral pre-SMA, bilateral dorsal aspect of M1, contralesional S1, contralesional IPS, and bilateral anterior cingulate cortex (ACC) emerged (recovery in Fig. 5*C* and Supplementary Fig. 5*D*). Thus, during the recovery stage, brain regions showing overlapping connectivity with the co-M1v and co-VSt increased for both grip tasks, but less so for the whole finger grip task. These results indicate that, after SCI, the strengthened functional connectivity among the motor-related areas and its tight association with the VSt may play a crucial role during the recovery course of precision grip.

### Effect of VSt Lesion on Finger Movements

Results in the preceding section of the current study suggest that the VSt may be a key node in the circuits responsible for functional recovery of dexterous finger movements. Previously, we demonstrated the direct involvement of the VSt in motor control during the early recovery stage after a partial SCI (Sawada et al. 2015). However, a causal contribution of the VSt to the functional recovery of finger movements has not been demonstrated. Consequently, we compared the recovery course of finger movements in VSt-lesioned and temporary inactivated monkeys following SCI (Fig. 1*B*). To assess finger dexterity, six macaque monkeys were trained in the slit task, as described in Materials and Methods, before the VSt intervention (Fig. 1*B*).

**Figure 6:**
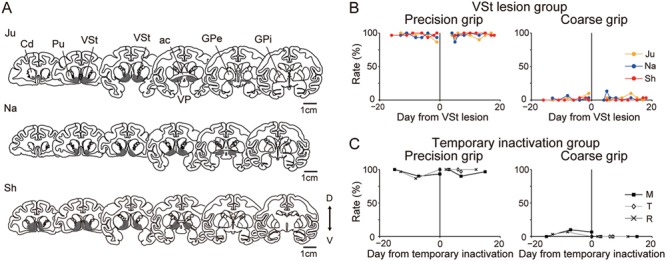
The effect of the VSt intervention on finger dexterity before SCI. (*A*) The extent of the VSt lesion. Six representative coronal sections through the VSt are arranged rostro-caudally for each monkey (Monkeys Ju, Na, and Sh). The gray hatch indicates the lesion area. Cd, caudate nucleus; ac, anterior commissure; GPe, external globus pallidus; D, dorsal; V, ventral. (*B*) Rates of the precision grip and coarse grip before and after the VSt lesion in the VSt lesion group (*N* = 3; Monkeys Ju, Na, and Sh). The rate (%) of precision grip and that of coarse grip were calculated as the number of successful trials with either grip divided by 30 trials, respectively. (*C*) Rates of the precision grip and coarse grip before and after the muscimol injection into the VSt in the temporary inactivation group (*N* = 3; Monkeys M, T, and R).

After training for the slit task for >6 weeks and achieving a stable performance of precision grip, the monkeys received either a permanent or a temporary inactivation of the VSt. Since brain imaging results showed that the activity of the co-M1v was correlated with that of bilateral VSt during the course of recovery of precision grip (recovery in Fig. 4*C*), we performed a permanent lesion of the VSt bilaterally by local injection of the neurotoxin ibotenic acid in three monkeys (VSt lesion group; Monkeys Ju, Na, and Sh). In the VSt lesion group, the lesion area in all three monkeys covered the entire extent of the bilateral VSt. The lesions extended into the ventral pallidum (VP) and the most ventral parts of both the caudate nucleus and putamen (Fig. 6*A*). After the bilateral VSt lesion, the monkeys remained in an unconscious state for 1–2 days after surgery, as reported previously (Stern and Passingham 1994, 1996). After recovery of consciousness, general behaviors, such as food intake and behavioral activity in their home cage, and general motivation to obtain a morsel of food were similar to those observed prior to the VSt lesion. In order to demonstrate the significance of a permanent lesion of the VSt on recovery, subjects in temporary inactivation group (Monkeys M, T, and R) received a temporary blockade of the bilateral VSt before SCI by injecting muscimol, a GABA_A_ receptor agonist, into the bilateral VSt. Our experience in the inactivation of motor cortices (Nishimura et al. 2007a; Tsuboi et al. 2010; Murata et al. 2015) or the VSt (Sawada et al. 2015) in monkeys using muscimol shows that the effect of muscimol disappears completely by 2 days after administration. After sufficient recovery from the VSt lesion (4–5 days) or temporary inactivation (~2 days) surgery, the monkeys were then trained and tested in the slit task for >2 weeks. None of the monkeys of either the VSt lesion or the temporary inactivation groups showed an impairment of finger dexterity (precision grip in Fig. 6*B*,*C* and Supplementary Video 1).

**Figure 7:**
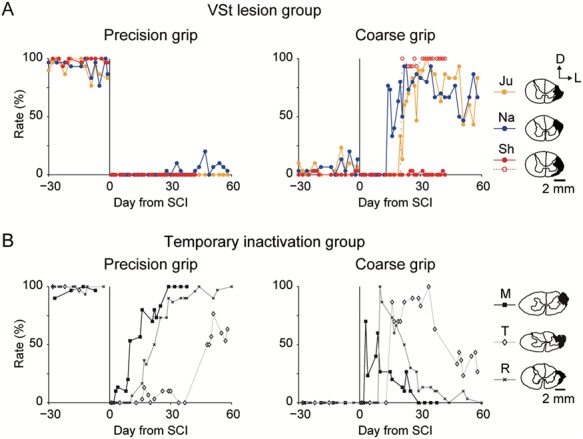
Recovery course of finger movements after SCI in the VSt intervention experiments. (*A*) Rates of the precision grip and coarse grip before and after SCI in the VSt lesion group (*N* = 3; Monkeys Ju, Na, and Sh). Monkey Sh showed a different strategy that did not satisfy our criteria of the coarse grip (red-filled circles, see Result). Red open circles in coarse grip graph show percentage of retrieving with a coarse grip including grasping the dropped food. (*B*) Rates of the precision grip and coarse grip before and after SCI in the temporary inactivation group (*N* = 3; Monkeys M, T, and R). Illustrations of coronal spinal cord sections on the right columns in (*A*) and (*B*) indicate lesion extent (black hatch) of the l-CST in individual monkeys. Illustrations of the temporary inactivation group were reprinted with permission from AAAS (Sawada et al. 2015). D, dorsal; L, lateral.

Monkeys in both groups were subjected to unilateral lesion of the l-CST at the border between the C4 and C5 segments. The lesion areas of the spinal cord were comparable in both groups (right column of Fig. 7*A* for the VSt lesion group and Fig. 7*B* for the temporary inactivation group). The monkeys in both groups showed decreased motor activity in general after SCI. However, none of the monkeys showed reduced motivation for food intake after SCI. All the monkeys began rehabilitative training for food retrieval with the lesion-affected hand on the day following the SCI with restriction of the lesion-unaffected hand. Precision grip was impaired in both groups immediately after the lesion (precision grip in Fig. 7*A*,*B*). In the temporary inactivation group, successful coarse grips were observed immediately after SCI. These coarse grips were replaced gradually by precision grips during the course of functional recovery (Fig. 7*B*). Precision grips recovered to pre-SCI levels within 60 days of daily training (precision grip in Fig. 7*B* and Supplementary Video 2). This finding is consistent with the results of the PET experiments (Fig. 2) and previous studies (Nishimura et al. 2007a, 2009, 2011; Higo et al. 2009; Sugiyama et al. 2013; Sawada et al. 2015; Tohyama et al. 2017). In contrast, recovery of precision grip in the VSt lesion group was impaired significantly compared with that of the temporary inactivation group, even though subjects received continuous daily rehabilitative training for 2 months (precision grip in Fig. 7*A* and Supplementary Videos 3-5). However, the VSt-lesioned monkeys recovered coarse grip gradually at 2–3 weeks after SCI (coarse grip in Fig. 7*A*), and use of this grip strategy lasted until the end of the period of observation (2 months). Two monkeys (Monkeys Ju and Na) raked the morsel out of the slit using a single index finger and then held it with a clenched hand without dropping it (Supplementary Videos 3 and 4). Similar observations were reported on the SCI model monkeys that did not receive early rehabilitation training during the 1-month after SCI (Sugiyama et al. 2013). Monkey Sh remained unable to retrieve the morsel from the narrow vertical slit and also showed particularly low rates of coarse grip (red-filled circles in coarse grip in Fig. 7*A*). However, Monkey Sh recovered coarse grip gradually by developing an alternative strategy of raking and dropping the morsel using his index finger, and then grasping it (Supplementary Video 5). Because the target was dropped, this new strategy did not match our criteria for successful precision grip or coarse grip (see details in Materials and Methods). Consequently, the rates of both precision grip and coarse grip remained low in Monkey Sh (red-filled circles in Fig. 7*A*). However, we confirmed that the skill of Monkey Sh in coarse grip recovered because he could grasp the dropped morsel with a coarse grip (red open circles in coarse grip in Fig. 7*A*).

## Discussion

The results of this study demonstrate that, during recovery from SCI, the VSt is associated more closely with motor-related networks in the execution of precision grip than whole finger grip. Furthermore, while permanent lesion of the VSt did not affect precision grip before SCI, it impeded the recovery of precision grip after SCI, confirming a causal contribution of the VSt to the functional recovery of finger dexterity after SCI. These results suggest that the VSt has a critical modulatory action and provides an essential contribution to the recovery of dexterous finger movements by facilitating the neuroplastic reorganization of motor-related networks after SCI.

### The VSt is Essential for Training-Dependent Recovery of Dexterous Finger Movements 

The results of the PET study showed that, during recovery from SCI, the activity of the VSt increased (Fig. 3*A*), and this activity was associated tightly with extensive motor-related networks only when conducting the precision grip, but not the whole finger grip after SCI (Fig. 5). Furthermore, results of our previous study (Sawada et al. 2015) demonstrated that, during the early recovery stage after SCI, reversible inactivation of the VSt caused a transient deficit of amelioration in finger dexterity obtained by rehabilitation. The same manipulation caused no comparable deficit during the pre-SCI period or in the late recovery stage. Those findings suggest that the VSt is critical for dexterous finger control during the early recovery stage (Sawada et al. 2015). VSt-lesioned monkeys in the present study showed impaired precision grip from the outset of SCI, similar to our previous results (Sawada et al. 2015). However, this impairment remained throughout the observation period during which the precision grip was completely recovered in the temporary inactivation group (precision grip in Fig. 7*A*,*B*). This result suggests that during early recovery stage, the VSt is essential for the recovery of finger dexterity beyond the control of dexterous finger movement.

In contrast to the time course of recovery for precision grip, coarse grip was recovered in the monkeys with temporary inactivation of VSt and those with permanent lesion of VSt, suggesting little effect of the VSt on reorganization of the motor-related network required for functional recovery of coarse grip. This was confirmed by our present results showing no increase in VSt activity throughout the recovery period of whole finger grip (Fig. 3*C*).

### Reorganization of the VSt-Motor Network for Functional Recovery of Dexterous Finger Movements

Early rehabilitative training after SCI (Winchester et al. 2005; Sugiyama et al. 2013) or brain injury (Biernaskie et al. 2004) shows a positive influence on subsequent functional recovery. Repetitive training of manual dexterity induces neuronal plasticity such as expansion of digit representation in the M1 (Nudo et al. 1996). Transient increases in the expression of plasticity-related molecules, have been reported in sensorimotor cortices (SMC), including the M1, PMv, and S1, during the early recovery stage after SCI (Higo et al. 2009, 2018). Furthermore, during the early recovery stage after SCI, the VSt up-regulates activity of the SMC and controls the dexterous finger movements directly (Sawada et al. 2015). These cortical-reorganizations associated with behavioral improvements after SCI occurs during the early recovery stage specifically. PET results in the present study also demonstrate the emergence of functional linkages among the VSt and extensive motor-related areas during recovery of precision grip (recovery in Fig. 5*B* and Supplementary Fig. 4*D*). Together, these findings indicate that an early start of rehabilitative training is critical for inducing plastic change in motor-related areas and promoting functional recovery. The VSt in this network could possibly drive the reorganization within the wide range of related neural networks required for the recovery of dexterous movements after SCI. Hence, during the early recovery stage, a lack of input to motor-related areas from the lesioned VSt may disrupt the reorganization required in the motor-related network for functional recovery of dexterous finger movements. Furthermore, CST lesion results in a lost connection between the motor-related cortical areas and target spinal motoneurons. Subjects were required to find residual pathways to spinal motoneurons and relearn a novel causal input–output relationship to control the affected limb. Such reorganization and adaptation for functional recovery following SCI may be represented by reorganization of the functional connectivity among VSt-motor networks (recovery in Fig. 5*B*). Thus, our results imply that the VSt, itself, and/or VSt-motor networks may be critical for the relearning of precision grip.

It is important to consider how the VSt and M1 increased concurrently the functional connectivity with the same brain areas in the motor-related network. Mogenson et al. (1980) proposed that the VSt may be a critical node in the limbic-motor interface involved in translation from motivation into action. The VSt receives inputs from the limbic systems (e.g., OFC) involved in the processing of motivation (Haber et al. 1995). It then projects via the VP to thalamocortical- and subcortical-motor-related networks (Alexander et al. 1983; Haber et al. 1990; Kelly and Strick 2004). The M1 directly and/or indirectly receives abundant projections from extensive frontal cortical and subcortical areas including the VSt (Miyachi et al. 2005). Therefore, the anatomical architecture is in place to allow the VSt to affect activity, concurrently with the M1 (Supplementary Fig. 5), in a range of sensorimotor-related areas (Fig. 5 and Supplementary Fig. 4), which are required for the recovery of dexterous finger movements after SCI.

### Methodological Consideration

There are two methodological issues in the present study, the lesion of CST and the neurotoxic lesion of the VSt, which should be considered.

In the present study, we performed a l-CST lesion at the upper cervical cord. Because dexterous finger movements depend largely on the CST in higher primates (Lawrence and Kuypers 1968a, 1968b; Nishimura et al. 2007a, 2007b, 2009, 2011; Higo et al. 2009, 2018; Sugiyama et al. 2013; Sawada et al. 2015), our approach of lesioning the l-CST results in severe impairment of dexterous movements. On the other hand, coarse movements are relatively unaffected by the l-CST lesion, as shown in the present study and previous studies (Nishimura et al. 2007a, 2007b; Sugiyama et al. 2013), suggesting a contribution of the residual pathways in the cervical cord, such as reticulospinal and/or propriospinal pathways, in coarse movements. When a larger lesion is performed, such as spinal hemisection or lesioning all descending and ascending pathways, the VSt might be involved in the recovery process of coarse grip, as well. Further studies are needed to investigate this hypothesis.

Second, we used the neurotoxin ibotenic acid to lesion the VSt. The neurotoxic lesion of the VSt destroyed neurons throughout the structure bilaterally, which established a model to examine the potential role of the VSt in the recovery of dexterous motor control. Ibotenic acid is used widely for the selective destruction of cell bodies, while allowing large myelinated fiber bundles to remain unharmed (Schwarcz et al. 1979; Köhler and Schwarcz 1983). However, a high dose of ibotenic acid damages both cell bodies and myelinated fiber bundles (Frey et al. 1997). Thus, it is difficult to evaluate whether the injection volume and concentration of ibotenic acid used in the present study selectively destroyed only cell bodies. If axons, such as input fibers or output fibers of the VSt, were also lesioned in the present study, axon degeneration would be propagated retrogradely or anterogradely from the VSt, which would cause the VSt to lose function as a hub that connects VSt-related networks. The VSt lesion may impede emergence of the VSt-motor network as demonstrated in the PET experiments (Fig. 5*B* and Supplementary Fig. 4*D*). In addition, in all cases, the VSt lesion extended to the VP (Fig. 6*A*). However, since the VP is a principal target structure of the VSt (Haber et al. 1990; Hedreen and DeLong 1991), we consider that the extension of the VSt lesion to the VP does not affect the main conclusions of this study for the same reason as described above.

### Clinical Implications

We demonstrated a causal contribution of the VSt in precise motor control and its recovery after SCI. The VSt is accepted widely as an important node for processing motivation to obtain reward. Results of several human and animal studies have suggested a critical involvement of the VSt in motivation-driven effort (Aberman and Salamone 1999; Pessiglione et al. 2007; Schmidt et al. 2012). Brodal described that the expenditure of “mental effort” depended on the severity of paralysis after neuronal injury (Brodal 1973). Thus, the VSt may provide the ‘mental effort’ required to motivate subjects to engage in the behavior needed for sensorimotor networks to retrain themselves for precise limb control made difficult by neuronal injury. Moreover, clinical studies have shown that 27–63.9% of patients with SCI (Krause et al. 2000; Wang et al. 2005; Anderson et al. 2007; Hassanpour et al. 2012; Shin et al. 2012) and 30–60% of patients with stroke (Morris et al. 1990; Saxena et al. 2007) show depressive symptoms. In addition, several studies have revealed that depressive states are associated with severity of residual motor function and functional recovery (Chemerinski et al. 2001; Saxena et al. 2007). It has also been noted that patients with depression (Cléry-Melin et al. 2011) or Parkinson’s disease (Chong et al. 2015) display preference for less-effort movements. These results suggest that improving the depressive mental status after neuronal damage is likely to facilitate functional recovery. Therefore, up-regulation of the VSt is likely to drive not only the reorganization of brain networks required for sensorimotor recovery, but also improve patients’ psychological wellbeing when they recognize that recovery is possible. In the light of these considerations, future studies should seek to identify psychological approaches that can activate the VSt (e.g., the use of strong positive reinforcement when patients attempt to be engaged in the impaired behavior), which may promote effective rehabilitation.

## Supplementary Material

SupplementaryVideo1_Suzuki_et_al_bhz307

SupplementaryVideo2_Suzuki_et_al_bhz307

SupplementaryVideo3_Suzuki_et_al_bhz307

SupplementaryVideo4_Suzuki_et_al_bhz307

SupplementaryVideo5_Suzuki_et_al_bhz307

SupplementaryMaterials_Suzuki_et_al_Final_191116_bhz307

## Notes

We thank P. Redgrave for comments on the manuscript and Y. Itani, M. Togawa, T. Eto, and K. Isa for technical help. Correspondence and requests for materials should be addressed to Y.N. (nishimura-yk@igakuken.or.jp). The authors declare no conflicts of interest.
