# Supplementary material for: The Ventral Striatum is a Key Node for Functional Recovery of Finger Dexterity After Spinal Cord Injury in Monkeys
Source: Cereb Cortex. 2019 Dec 9;30(5):3259–70. doi: 10.1093/cercor/bhz307 (PMC7197201; doi:10.1093/cercor/bhz307)
Supplement: SupplementaryMaterials_Suzuki_et_al_Final_191116_bhz307 [file supplementarymaterials_suzuki_et_al_final_191116_bhz307.docx]

*Supplementary materials*

The ventral striatum is a key node for functional recovery of finger dexterity after spinal cord injury in monkeys

M. Suzuki, K. Onoe, M. Sawada, N. Takahashi, N. Higo, Y. Murata, H. Tsukada, T. Isa, H. Onoe, Y. Nishimura

This material includes abbreviations for supplementary figures and tables, 5 supplementary figures, 6 supplementary tables, and 5 supplementary video captions.

**Abbreviations for Supplementary Figures and Tables**

ACC, anterior cingulate cortex; Area 8, Brodmann area 8; Area 9, Brodmann area 9; Area 9m, medial part of Brodmann area 9; Area 14m, medial part of Brodmann area 14; Area 14o, orbital part of Brodmann area 14; Area 24, Brodmann area 24; Area 46, Brodmann area 46; Area 46v, ventral part of Brodmann area 46; BW, body weight; cACC, caudal anterior cingulate cortex; Cb, cerebellum; Cd, caudate nucleus; CG, cingulate gyrus; Cl, claustrum; contra, contralesional; DpMe, deep mesencephalic nucleus; GP, globus pallidus; GPe, external globus pallidus; GPi, internal globus pallidus; l-CST, lateral corticospinal tract; LIP, lateral intraparietal area; M1d, dorsal aspect of the primary motor cortex; M1v, ventral aspect of the primary motor cortex; co-M1v, contralesional-M1v; MIP, medial intraparietal area; MT, middle temporal visual area; MTG, middle temporal gyrus; Ins, Insular cortex; IPS, intraparietal sulcus; ipsi, ipsilesional; ITG, inferior temporal gyrus; OFC, orbitofrontal cortex; PaAC, caudal part of paraauditory cortex; PCC, posterior cingulate cortex; PE, parietal area PE; PECg, cingulate part of parietal area PE; PFG, parietal area PFG; PG, parietal area PG; PGM, medial part of parietal area PG; PGOp, opercular part of parietal area PG; PMd, dorsal premotor area; PMv, ventral premotor area; PPC, posterior parietal cortex; PPTg, pedunculopontine tegmental nucleus; pre-SMA, pre-supplementary motor area; Pu, putamen; rACC, rostral part of anterior cingulate cortex; rCBF, regional cerebral blood flow; RN, red nucleus; S1, somatosensory motor cortex; S2, secondary somatosensory cortex; SCI, spinal cord injury; SN, substantia nigra; SMA, supplementary motor area; ST1, superior temporal sulcus area 1; STG, superior temporal gyrus; TE1, temporal area TE1; TE2, temporal area TE2; TEa, temporal area TEa; TEO, occipital part of temporal area TE; TEOM, occipitomedial part of temporal area TE; Th, thalamus; TLR, rostral part of temporal area TL; TPO, temporal parieto-occipital associated area; TPPro, temporopolar proisocortex; V1, primary visual cortex; V2, visual area 2; V3, visual area 3; V3d, dorsal part of visual area 3; V3v, ventral part of visual are 3; V4d, dorsal part of visual area 4; V4v, ventral part of visual area 4; V6, visual area 6; VIP, ventral intraparietal area; VOI, voxels of interest; VP, ventral pallidum; VSt, ventral striatum; co-VSt, contralesional-VSt.

**
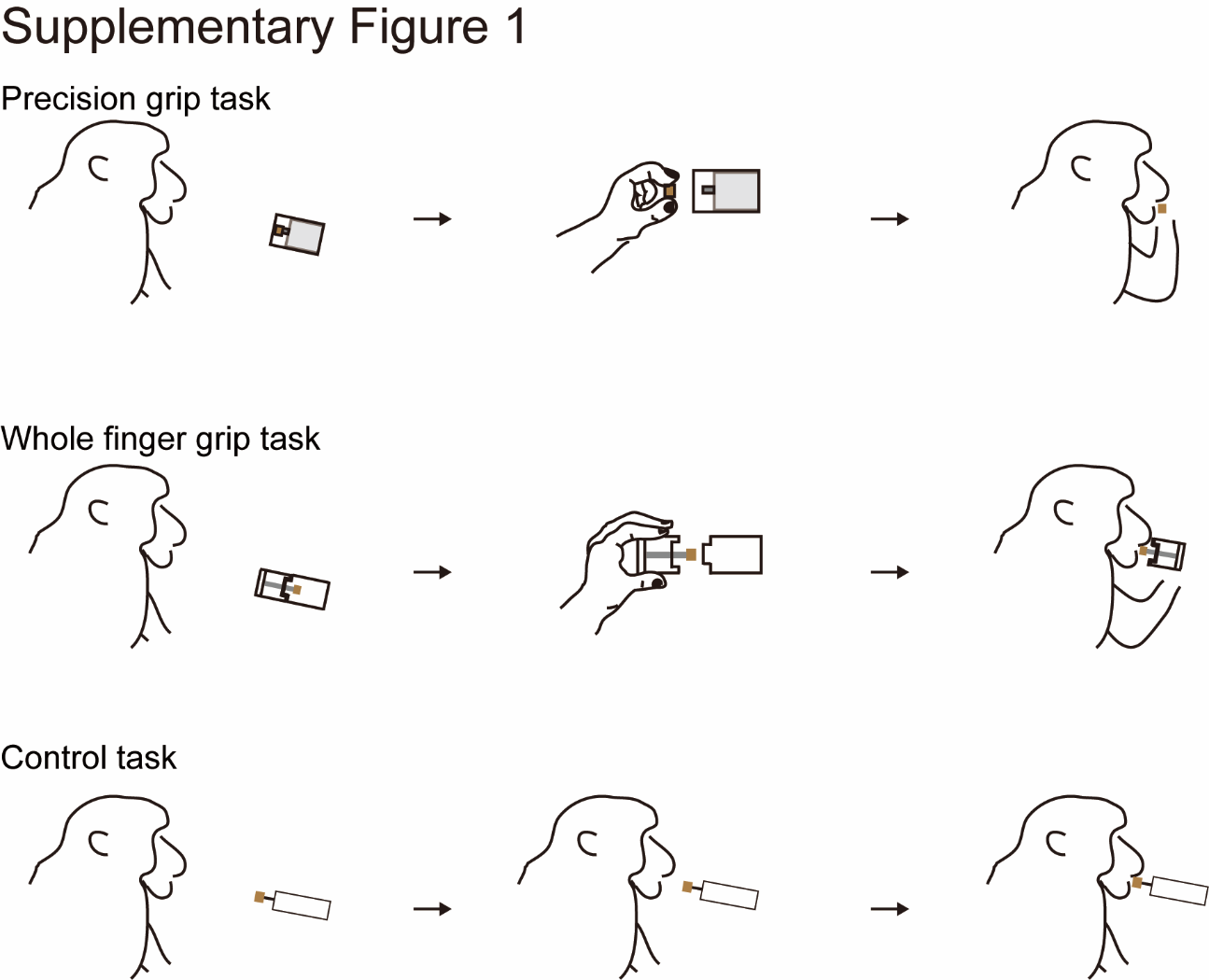
**

**Supplementary Figure 1.** **Behavioral tasks for the PET experiments.**

The monkeys were trained to sit on the monkey chair and to perform the precision grip task (Top), the whole finger grip task (Middle) and the control task (Bottom). In both of the precision and whole finger grip tasks, after successful grasping, the monkey ate the sweet potato. In the control task, a morsel of sweet potato was placed on the tip of a rod attached to a long tube that was presented directly to their mouth while both arms were restricted. The food morsel approached the monkeys from the same position and angle as in the precision grip/whole finger grip task. See also Materials and Methods for detailed description.

**
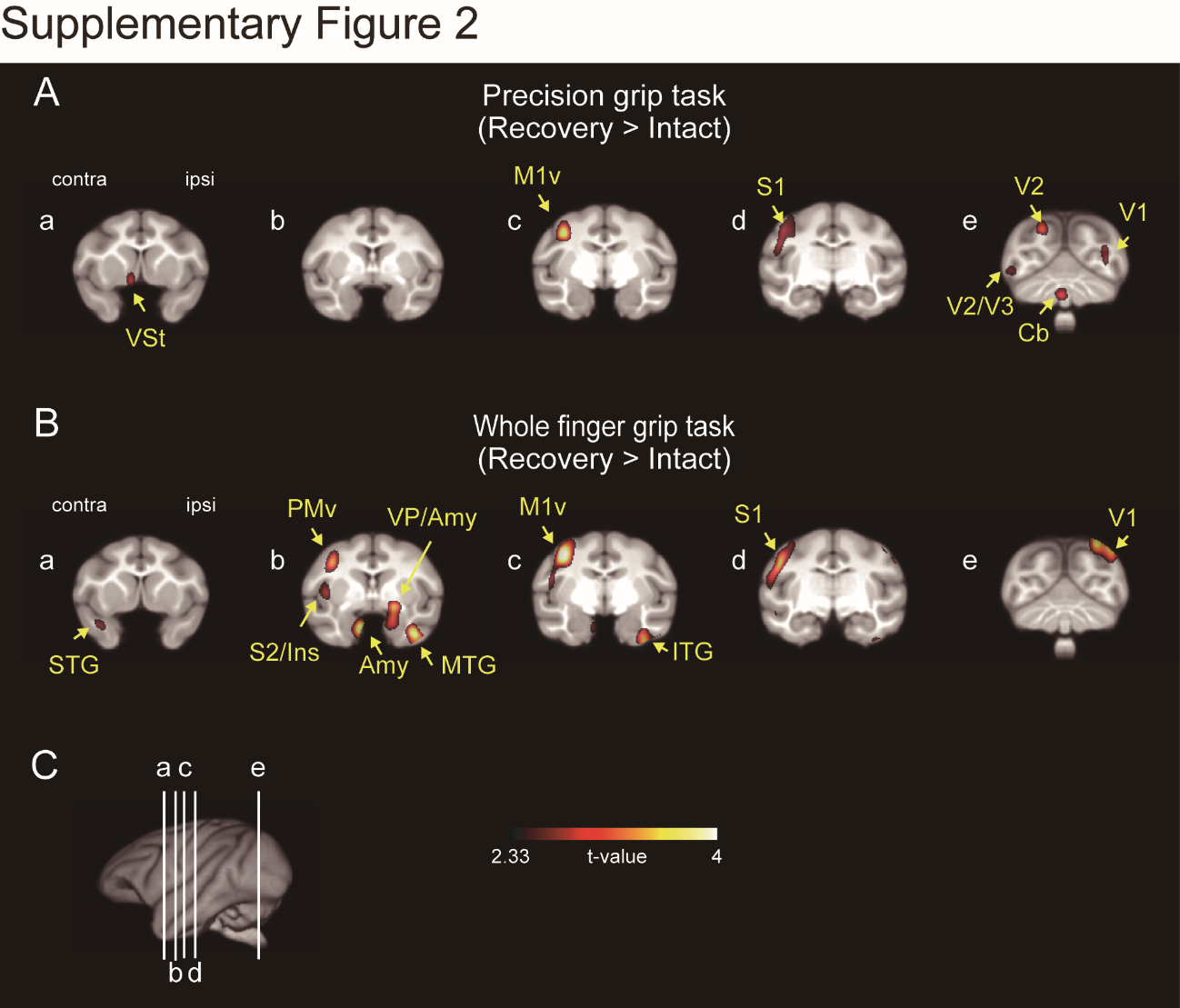
**

**Supplementary Figure 2. Increased brain activation related to functional recovery.**

Brain areas with significantly increased rCBF (*P*<0.01, uncorrected for multiple comparison) are superimposed on a template brain MRI of macaque monkeys. Results were obtained from the three monkeys and averaged. (A) Precision grip task. (B) Whole finger grip task. The significance level is provided in terms of t-values represented on a color scale. (C) Lines (a) - (e) indicate the levels of coronal sections of (a) - (e) in (A) and (B). See also Supplementary Table 4.


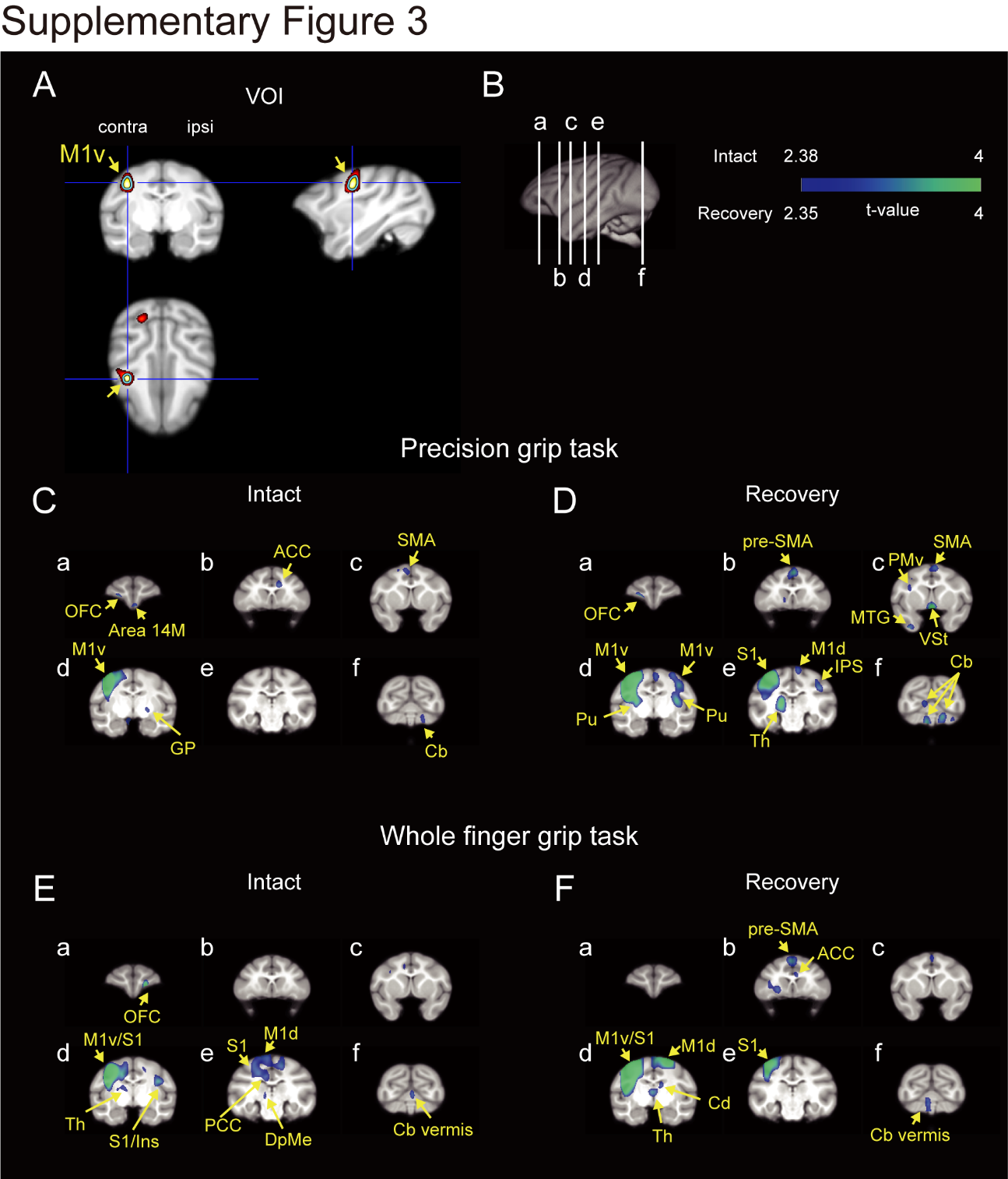


**Supplementary Figure 3. Functional connectivity of the contralesional M1 before and after SCI.**

(A) VOI in the ventral aspect of the co-M1v is outlined by cyan line. VOI of the co-M1v was the entire region of significant functional activation thresholded at *P*<0.001 (t>3.1) consisting of 720 voxels. The correlations were calculated between the rCBF value of the VOI in the co-M1v and that in other regions during the behavioral task. Brain areas that have significant positive correlation (*P*<0.01, uncorrected for multiple comparisons) are shown on a template brain MRI. (B) Lines (a) - (f) indicate the levels of coronal sections of (a) - (f) in (C) - (F). (C and D) Precision grip task (C) before and (D) after SCI. (E and F) Whole finger grip task (E) before and (F) after SCI. The significance level is given in terms of t-values represented in a colored scale. See also Supplementary Table 5.


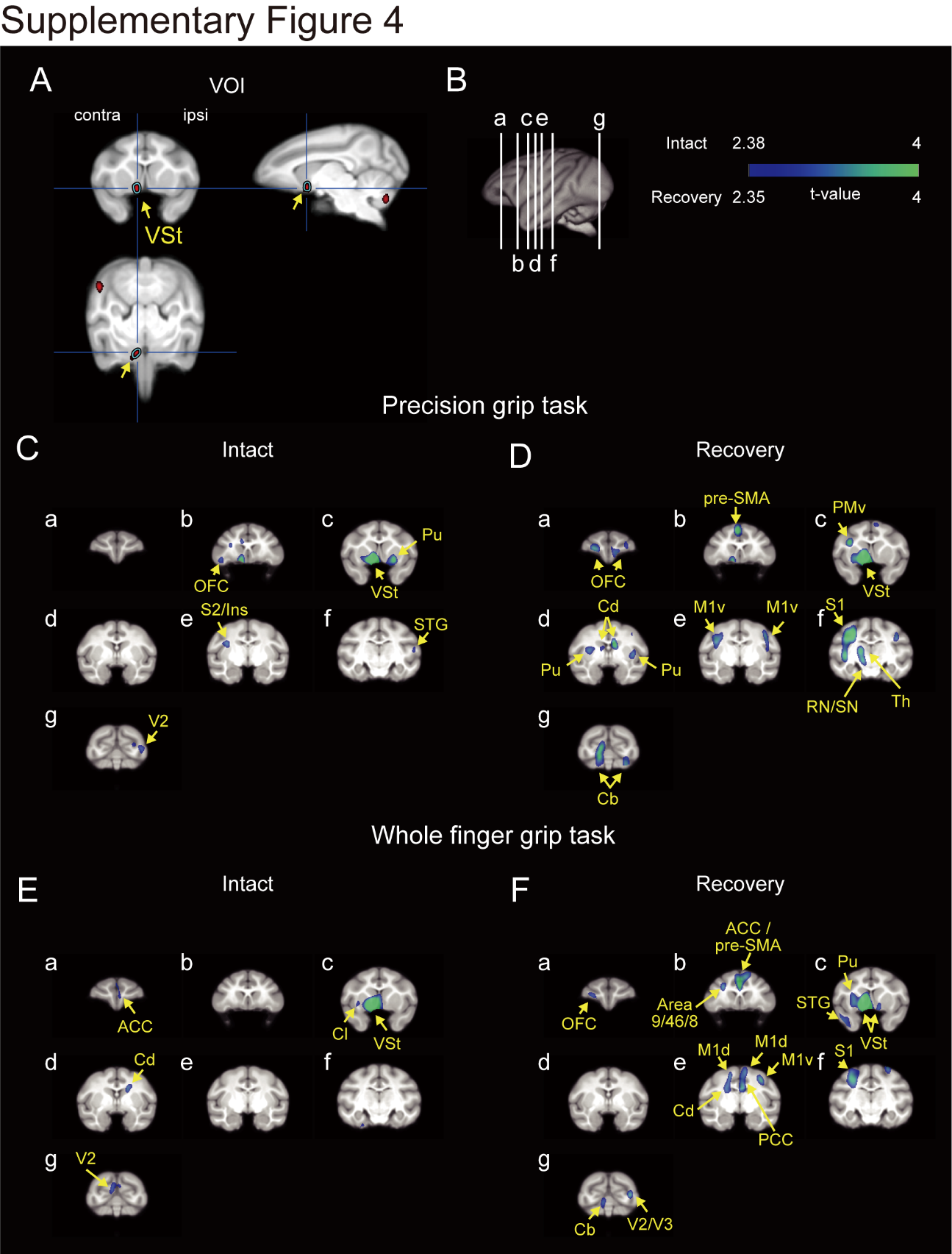


**Supplementary Figure 4. Functional connectivity with the contralesional VSt before and after SCI.**

(A) VOI of the co-VSt is outlined by cyan line. VOI of the co-VSt was the entire region of significant functional activation thresholded at *P*<0.01 (t>2.3) consisting of 190 voxels. The correlations were calculated between the rCBF value of the VOI in co-VSt and that in other regions during the behavioral task. Brain areas that have significant positive correlation (*P*<0.01, uncorrected for multiple comparisons) are indicated on a template brain MRI. (B) Lines (a) - (g) indicate the levels of coronal sections of (a) - (g) in (C) - (F). (C and D) Precision grip task (C) before and (D) after SCI. (E and F) Whole finger grip task (E) before and (F) after SCI. The significance level is provided in terms of t-values represented a colored scale. See also Supplementary Table 6.


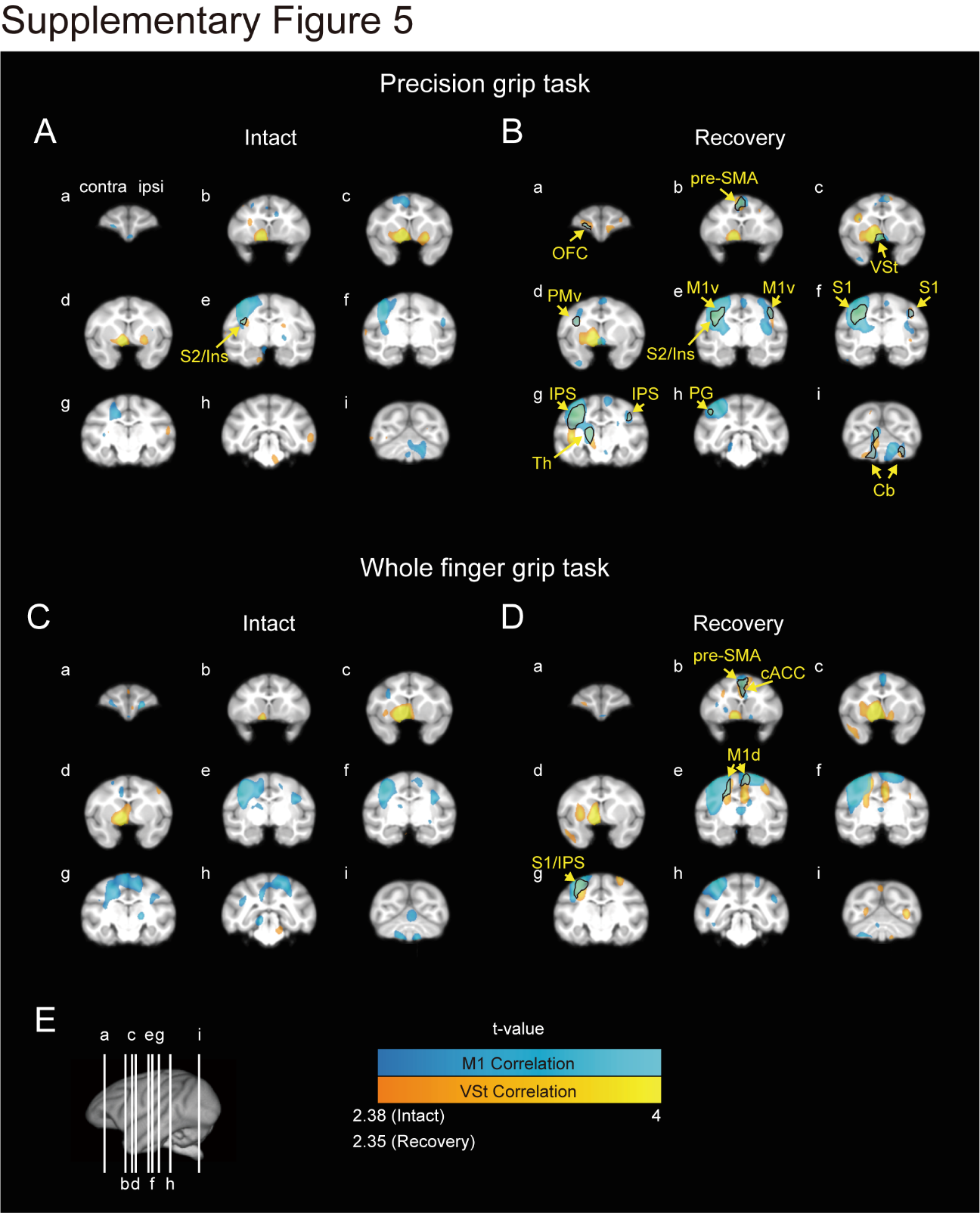


**Supplementary Figure 5. Overlapping functional connectivity between the co-VSt and co-M1.**

Brain regions revealing overlapping functional connectivity with the co-VSt and co-M1v are shown by black outlines. Brain regions with the co-VSt and co-M1v network are indicated by orange color and blue color, respectively. (A and B) Precision grip task: (A) before and (B) after SCI. (C and D) Whole finger grip task: (C) before and (D) after SCI. The significance level is provided in terms of t-values represented a colored scale. (E) Lines (a) - (i) indicate the levels of coronal sections of (a) - (i) in (A) - (D).

**Supplementary Table 1. Summary of subjects’ information**

| Experiment | Spinal cord | Bilateral VSt | Subject | Species | Sex | Age (y) | BW (kg) |
| --- | --- | --- | --- | --- | --- | --- | --- |
| PET | l-CST lesion | No intervention | H | *M. fuscata* | Female | 6.0 | 6.7 |
|  |  |  | K | *M. mulatta* | Male | 7.8 | 6.5 |
|  |  |  | TF | *M. mulatta* | Male | 7.8 | 8.1 |
| VSt intervention | l-CST lesion | Permanent lesion | Ju | *M. mulatta* | Female | 5.3 | 4.6 |
|  |  |  | Na | *M. mulatta* | Female | 5.5 | 4.6 |
|  |  |  | Sh | *M. mulatta* | Female | 7.5 | 3.5 |
|  |  | Temporary inactivation | M | *M. fuscata* | Male | 6.6 | 7.6 |
|  |  |  | T | *M. fuscata* | Female | 3.8 | 5.1 |
|  |  |  | R | *M. fuscata* | Female | 5.3 | 5.5 |

**Supplementary Table 2. Summary of data information in the PET experiments**

| Group | Subjects | Behavioral data in the pin task | | Behavioral data in the slit task | | PET data | | |
| --- | --- | --- | --- | --- | --- | --- | --- | --- |
|  |  | Precision grip | Coarse grip | Precision grip | Coarse grip | Precision grip task | Whole finger grip task | Control task |
| PET | Monkey H | Novel use | Novel use | Reuse  (Nishimura et al. 2007a, 2011) | Novel use | Reuse  (Nishimura et al. 2007a, 2007b, 2011) | Novel use | Reuse  (Nishimura et al. 2007a, 2007b, 2011) |
|  | Monkey K | Novel use | Novel use | Reuse  (Nishimura et al. 2007a, 2011) | Novel use | Reuse  (Nishimura et al. 2007a, 2007b, 2011) | Novel use | Reuse  (Nishimura et al. 2007a, 2007b, 2011) |
|  | Monkey TF | Novel use | Novel use | Reuse  (Nishimura et al. 2007a, 2011) | Novel use | Reuse  (Nishimura et al. 2007a, 2007b, 2011) | Novel use | Reuse  (Nishimura et al. 2007a, 2007b, 2011) |

**Supplementary Table 3. Summary of data information in the VSt intervention experiments**

| Group | Subjects | Behavioral data  before and after bilateral VSt intervention | | | Behavioral data after SCI | |
| --- | --- | --- | --- | --- | --- | --- |
|  |  | Precision grip | Coarse grip | Precision grip | | Coarse grip |
| Permanent lesion | Monkey Ju | Novel use | Novel use | Novel use | | Novel use |
|  | Monkey Na | Novel use | Novel use | Novel use | | Novel use |
|  | Monkey Sh | Novel use | Novel use | Novel use | | Novel use |
| Temporary inactivation | Monkey M | Novel use (Before),  Reuse (After)  (Sawada et al., 2015) | Novel use | Reuse  (Sawada et al., 2015) | | Novel use |
|  | Monkey T | Reuse  (Sawada et al., 2015) | Novel use | Reuse  (Sawada et al., 2015) | | Novel use |
|  | Monkey R | Novel use | Novel use | Reuse  (Sawada et al., 2015) | | Novel use |

**Supplementary Table 4. Statistical analysis of the rCBF increase related to functional recovery during the precision grip task and the whole finger grip task in the recovery stage compared with that in the intact stage.**

| **Precision grip**  Brain region | Laterality | t-value | x | y | z | Remarks |
| --- | --- | --- | --- | --- | --- | --- |
| **Recovery > Intact** |  |  |  |  |  |  |
| VSt | Contra | 2.894 | 86 | 72 | 44 |  |
| M1 (ventral) | Contra | 3.984 | 61 | 91 | 83 | Extend to S1 |
| S2 | Contra | 2.815 | 49 | 99 | 70 |  |
| V2 | Contra | 2.764 | 53 | 135 | 68 |  |
| V2/V3v | Contra | 3.136 | 47 | 141 | 48 |  |
| V2 | Contra | 3.209 | 75 | 149 | 87 |  |
| V1 | Ipsi | 3.014 | 128 | 148 | 66 |  |
| Cb vermis | Mid | 3.501 | 92 | 153 | 32 |  |
| **Whole finger grip**  Brain region | Laterality | t-value | x | y | z | Remarks |
| **Recovery > Intact** |  |  |  |  |  |  |
| TPPro | Contra | 2.76 | 63 | 76 | 30 |  |
| Amygdala | Contra | 3.843 | 84 | 83 | 27 |  |
| TE1/TE2 | Ipsi | 3.895 | 128 | 81 | 23 |  |
| VP/Amygdala | Ipsi | 3.684 | 111 | 80 | 42 |  |
| Insular/S2 | Contra | 3.064 | 56 | 80 | 54 |  |
| M1 (ventral) | Contra | 4.42 | 60 | 90 | 87 | Extend to PMv, S1 |
| S1 | Ipsi | 2.705 | 135 | 95 | 91 |  |
| S1 | Contra | 3.58 | 51 | 97 | 79 |  |
| S2 | Contra | 3.277 | 42 | 102 | 65 |  |
| TEa/TPO | Contra | 2.918 | 46 | 107 | 41 |  |
| PFG | Ipsi | 3.047 | 138 | 110 | 91 |  |
| PG (rostral) | Ipsi | 2.944 | 137 | 118 | 92 |  |
| PG (caudal) | Ipsi | 2.727 | 133 | 131 | 95 |  |
| TEOM | Contra | 2.828 | 43 | 123 | 70 |  |
| TEO | Contra | 3.344 | 40 | 128 | 43 |  |
| V2/V3v | Contra | 2.781 | 49 | 141 | 59 | Extend to V1 |
| V1 | Ipsi | 3.715 | 119 | 151 | 96 |  |

The level of the coefficients was set at *P*<0.01 (t>2.33). t-values at the center of individual masses of activation (the locations are indicated with the positions along the x-, y- and z-axis) are indicated. See also Supplementary Figure 2.

**Supplementary Table 5. Statistical analysis of the correlation of the rCBF in the co-M1v with that in other brain regions during the intact and the recovery stages.**

| **Precision grip**  Brain region | Laterality | t-value | x | y | z | Remarks |
| --- | --- | --- | --- | --- | --- | --- |
| **Intact** |  |  |  |  |  |  |
| Area 14m | Ipsi | 3.427 | 97 | 44 | 45 |  |
| OFC | Contra | 4.017 | 74 | 42 | 57 |  |
| rACC | Contra | 3.243 | 89 | 47 | 57 |  |
| Area 46 | Contra | 3.217 | 69 | 52 | 72 |  |
| cACC | Ipsi | 2.981 | 104 | 62 | 73 |  |
| PMd | Contra | 2.981 | 74 | 70 | 90 |  |
| SMA | Contra | 3.401 | 89 | 72 | 90 |  |
| Amygdala | Contra | 3.112 | 88 | 90 | 31 |  |
| Globus pallidus | Ipsi | 3.374 | 113 | 87 | 47 |  |
| M1 (ventral) | Contra | 37.59 | 60 | 92 | 84 | Extend to S1, S2. Insular |
| S2 | Ipsi | 3.073 | 138 | 98 | 68 |  |
| Putamen | Contra | 3.578 | 63 | 99 | 59 |  |
| PCC | Contra | 3.468 | 80 | 104 | 82 |  |
| V1 | Ipsi | 2.945 | 128 | 135 | 61 |  |
| Cb | Ipsi | 3.615 | 125 | 130 | 34 |  |
| Cb | Ipsi | 3.991 | 100 | 141 | 21 |  |
| Cb | Ipsi | 2.615 | 110 | 147 | 26 |  |
| Cb | Mid | 2.798 | 95 | 149 | 45 |  |
| Cb | Ipsi | 3.318 | 110 | 154 | 37 |  |
| Cb vermis | Mid | 2.78 | 92 | 155 | 30 |  |
|  |  |  |  |  |  |  |
| **Recovery** |  |  |  |  |  |  |
| OFC | Contra | 3.972 | 74 | 36 | 62 |  |
| Pre-SMA | Mid | 3.549 | 95 | 64 | 91 |  |
| SMA | Mid | 3.145 | 97 | 77 | 98 |  |
| SMA/M1 (medial) | Mid | 3.113 | 96 | 90 | 95 |  |
| VSt | Mid | 4.54 | 95 | 74 | 42 |  |
| PMv | Contra | 2.594 | 64 | 75 | 68 |  |
| TE1 | Contra | 3.291 | 63 | 75 | 15 |  |
| Putamen | Ipsi | 3.619 | 126 | 91 | 60 |  |
| Putamen | Contra | 4.224 | 70 | 95 | 63 |  |
| Insular | Ipsi | 2.515 | 134 | 97 | 58 |  |
| M1 (dorsal) | Ipsi | 3.488 | 123 | 89 | 93 |  |
| M1 (ventral) | Ipsi | 3.362 | 129 | 91 | 83 |  |
| S1 | Ipsi | 3.396 | 129 | 105 | 80 |  |
| M1 (dorsal) | Ipsi | 3.016 | 102 | 107 | 103 |  |
| M1 (ventral) | Contra | 58.72 | 60 | 91 | 83 | Extend to S1, PPC, S2, Insular |
| Thalamus | Contra | 4.5 | 79 | 106 | 61 |  |
| MIP | Ipsi | 3.632 | 103 | 130 | 95 |  |
| LIP/MIP | Contra | 3.041 | 71 | 129 | 92 |  |
| V4v/V3v | Contra | 4.277 | 57 | 131 | 47 |  |
| Cb | Contra | 3.199 | 79 | 122 | 43 |  |
| Cb | Ipsi | 4.737 | 107 | 153 | 40 |  |
| Cb | Contra | 4.25 | 82 | 147 | 32 |  |
| Cb vermis | Contra | 3.724 | 87 | 152 | 61 |  |
| Cb | Ipsi | 2.954 | 123 | 156 | 32 |  |
| **Whole finger grip**  Brain region | Laterality | t-value | x | y | z | Remarks |
| **Intact** |  |  |  |  |  |  |
| OFC | Ipsi | 4.187 | 111 | 39 | 58 |  |
| PMv | Contra | 3.112 | 67 | 72 | 72 |  |
| Putamen | Ipsi | 3.099 | 111 | 81 | 60 |  |
| S2/Insular | Ipsi | 3.571 | 126 | 97 | 73 | Extend to S1 |
| SMA | Ipsi | 3.037 | 103 | 85 | 91 |  |
| cACC/SMA | Contra | 4.554 | 79 | 88 | 84 |  |
| M1 (ventral) | Contra | 36.3 | 60 | 92 | 84 | Extend to S1, S2, Insular |
| S1 | Contra | 3.761 | 74 | 104 | 85 | Extend to VIP, MIP, LIP. AIP |
| S2/Insular | Contra | 3.495 | 56 | 101 | 74 |  |
| M1 (dorsal) | Ipsi | 3.5 | 107 | 103 | 94 |  |
| Thalamus | Ipsi | 3.771 | 110 | 104 | 54 |  |
| Thalamus | Contra | 2.737 | 83 | 94 | 62 |  |
| PCC | Contra | 3.972 | 85 | 115 | 79 |  |
| PE/MIP | Ipsi | 3.716 | 113 | 117 | 96 |  |
| DpMe | Contra | 3.881 | 82 | 117 | 48 |  |
| PaAC | Contra | 2.863 | 59 | 119 | 72 |  |
| TPO | Contra | 2.734 | 60 | 128 | 73 |  |
| PGM | Ipsi | 3.068 | 99 | 128 | 89 |  |
| V2 | Contra | 3.378 | 79 | 147 | 68 |  |
| Cb | Ipsi | 2.963 | 97 | 125 | 50 |  |
| Cb | Ipsi | 4.213 | 124 | 136 | 35 |  |
| Cb | Ipsi | 4.187 | 104 | 148 | 25 |  |
| Cb | Contra | 3.835 | 84 | 150 | 25 |  |
| Cb | Mid | 3.236 | 97 | 155 | 56 |  |
| Cb vermis | Mid | 4.436 | 97 | 168 | 49 |  |
|  |  |  |  |  |  |  |
| **Recovery** |  |  |  |  |  |  |
| OFC | Contra | 3.79 | 70 | 57 | 49 |  |
| cACC | Ipsi | 2.95 | 99 | 66 | 71 |  |
| Pre-SMA | Mid | 4.09 | 96 | 69 | 91 |  |
| Caudate | Ipsi | 2.84 | 104 | 92 | 68 |  |
| Thalamus | Mid | 3.3 | 93 | 92 | 57 |  |
| M1 (dorsal) | Ipsi | 5.436 | 114 | 89 | 98 |  |
| M1 (ventral) | Contra | 53.86 | 60 | 91 | 84 | Extend to PM/, S1, PPC, S2, Insular |
| M1 (medial) | Mid | 3.368 | 94 | 89 | 94 |  |
| PG/PE/LIP/MIP | Contra | 2.701 | 47 | 119 | 86 |  |
| MT | Contra | 3.051 | 50 | 119 | 60 |  |
| PGOp/PaAC | Ipsi | 3.134 | 136 | 118 | 77 |  |
| PE/MIP | Ipsi | 2.867 | 115 | 116 | 94 |  |
| Cb | Contra | 3.074 | 87 | 137 | 55 |  |
| Cb | Contra | 4.513 | 69 | 145 | 27 |  |
| Cb vermis | Contra | 2.951 | 89 | 156 | 44 |  |

The level of the coefficients was set at *P*<0.01 (t>2.38 for the intact stage, t>2.35 for the recovery stage). t-values at the center of individual masses of correlation (the locations are indicated with the positions along the x-, y- and z-axis) are indicated. See also Supplementary Figure 3.

**Supplementary Table 6. Statistical analysis of the correlation of the rCBF in the co-VSt with that in other brain regions during the intact and the recovery stages.**

| **Precision grip**  Brain region | Laterality | t-value | x | y | z | Remarks |
| --- | --- | --- | --- | --- | --- | --- |
| **Intact** |  |  |  |  |  |  |
| OFC | Ipsi | 3.688 | 122 | 52 | 59 |  |
| OFC | Contra | 4.751 | 56 | 54 | 49 |  |
| rACC | Contra | 3.309 | 88 | 55 | 64 |  |
| Area 45 | Contra | 2.902 | 69 | 64 | 65 |  |
| Putamen/VSt | Ipsi | 4.109 | 115 | 73 | 45 |  |
| VSt | Contra | 32.35 | 85 | 72 | 43 | Extend to Putamen |
| S2/insular | Contra | 3.083 | 63 | 91 | 70 |  |
| TPO | Ipsi | 3.239 | 148 | 110 | 61 |  |
| Pontine nuclei | Ipsi | 2.872 | 102 | 119 | 26 |  |
| TEO | Ipsi | 2.908 | 147 | 120 | 51 |  |
| V4d | Contra | 3.184 | 49 | 130 | 67 |  |
| V2/V3d | Ipsi | 2.954 | 143 | 135 | 65 |  |
| V1/V2 | Ipsi | 2.936 | 143 | 142 | 73 |  |
| V3d/V4d | Ipsi | 2.624 | 132 | 144 | 75 |  |
| V1 | Contra | 3.037 | 48 | 148 | 51 |  |
| V1 | Ipsi | 2.587 | 119 | 162 | 67 |  |
| V1/Cb vermis | Mid | 3.376 | 96 | 164 | 61 |  |
| V2 | Ipsi | 2.927 | 129 | 161 | 60 |  |
| V1 | Ipsi | 2.642 | 114 | 167 | 69 |  |
| V1 | Contra | 3.44 | 87 | 172 | 62 |  |
|  |  |  |  |  |  |  |
| **Recovery** |  |  |  |  |  |  |
| Area 46 | Contra | 3.214 | 82 | 34 | 59 |  |
| OFC | Contra | 3.471 | 75 | 44 | 62 |  |
| OFC | Ipsi | 3.123 | 105 | 42 | 55 |  |
| Area 46v | Ipsi | 2.739 | 120 | 44 | 66 |  |
| Area 46v | Ipsi | 2.812 | 121 | 53 | 70 |  |
| rACC | Ipsi | 3.018 | 97 | 47 | 69 |  |
| Pre-SMA | Mid | 4.632 | 91 | 63 | 88 |  |
| Putamen | Contra | 3.422 | 71 | 74 | 46 |  |
| PMv | Contra | 4.158 | 63 | 75 | 71 |  |
| VSt | Contra | 40.86 | 85 | 72 | 44 | Extend to Putamen |
| SMA | Ipsi | 2.765 | 100 | 72 | 95 |  |
| Caudate | Contra | 2.61 | 84 | 81 | 63 |  |
| Caudate | Ipsi | 4.31 | 102 | 84 | 70 |  |
| Putamen | Ipsi | 3.152 | 129 | 83 | 54 |  |
| Putamen | Contra | 3.158 | 66 | 84 | 61 |  |
| Insula/S2 | Ipsi | 3.226 | 132 | 86 | 58 |  |
| M1 (ventral) | Ipsi | 3.387 | 131 | 90 | 82 |  |
| S1 | Ipsi | 3.684 | 132 | 101 | 82 |  |
| S1 | Contra | 4.796 | 67 | 102 | 83 |  |
| M1 (ventral) | Contra | 2.608 | 61 | 91 | 83 | Extend to S2/Insular |
| RN/Reticular formation | Contra | 3.647 | 82 | 103 | 47 |  |
| Thalamus | Contra | 3.807 | 79 | 106 | 60 |  |
| TPO | Contra | 3.982 | 56 | 105 | 53 |  |
| PG/PGOp | Contra | 2.566 | 55 | 118 | 87 |  |
| TPO | Contra | 2.681 | 64 | 123 | 72 |  |
| MIP/LIP | Ipsi | 3.036 | 110 | 129 | 95 |  |
| MIP/LIP | Ipsi | 2.819 | 110 | 139 | 91 |  |
| MIP/LIP | Contra | 3.464 | 83 | 143 | 97 |  |
| V4v | Ipsi | 2.899 | 134 | 135 | 44 |  |
| V1 | Contra | 3.79 | 83 | 161 | 62 |  |
| V1 | Ipsi | 2.805 | 121 | 157 | 69 |  |
| V1 | Contra | 2.393 | 84 | 156 | 90 |  |
| Cb | Contra | 2.935 | 72 | 154 | 30 |  |
| Cb | Ipsi | 3.478 | 119 | 155 | 35 |  |
| Cb | Contra | 4.109 | 81 | 158 | 48 |  |
|  |  |  |  |  |  |  |
|  |  |  |  |  |  |  |
| **Whole finger grip**  Brain region | Laterality | t-value | x | y | z | Remarks |
| **Intact** |  |  |  |  |  |  |
| Area 14m/14o | Ipsi | 2.881 | 100 | 37 | 53 |  |
| Area 9m | Mid | 2.541 | 94 | 41 | 76 |  |
| rACC | Mid | 2.872 | 97 | 45 | 63 |  |
| Claustrum | Contra | 2.734 | 63 | 72 | 49 |  |
| VSt | Contra | 28.98 | 85 | 72 | 43 | Extend to Putamen, Caudate |
| Caudate | Ipsi | 3.147 | 110 | 83 | 73 |  |
| TLR | Contra | 3.009 | 70 | 102 | 18 |  |
| PPTg | Ipsi | 2.853 | 105 | 114 | 39 |  |
| V2 | Ipsi | 2.623 | 98 | 159 | 74 |  |
| V2 | Contra | 3.147 | 87 | 163 | 67 |  |
|  |  |  |  |  |  |  |
| **Recovery** |  |  |  |  |  |  |
| OFC | Contra | 2.879 | 69 | 43 | 61 |  |
| cACC | Mid | 4.276 | 94 | 62 | 76 | Extend to Pre-SMA |
| Area 9/46 | Contra | 3.259 | 70 | 62 | 74 |  |
| VSt | Contra | 39.91 | 85 | 72 | 44 | Extend to Caudate, Putamen |
| VSt | Ipsi | 3.18 | 106 | 72 | 43 |  |
| ST1/TPPro | Contra | 3.005 | 55 | 73 | 24 |  |
| Putamen | Contra | 3.834 | 67 | 78 | 52 |  |
| Caudate | Contra | 3.337 | 73 | 95 | 73 |  |
| M1 (ventral) | Ipsi | 3.512 | 125 | 91 | 86 | Extend to S1 |
| M1 (dorsal) | Ipsi | 3.102 | 101 | 91 | 97 |  |
| M1 (dorsal) | Contra | 3.76 | 80 | 94 | 92 |  |
| PCC | Mid | 3.862 | 98 | 95 | 80 |  |
| S1 | Ipsi | 3.572 | 118 | 107 | 103 |  |
| VIP/LIP/MIP | Contra | 4.086 | 69 | 111 | 83 |  |
| PECg | Contra | 2.866 | 79 | 113 | 80 |  |
| V4v | Ipsi | 3.196 | 134 | 133 | 42 |  |
| V6 | Contra | 3.203 | 92 | 148 | 88 |  |
| V2/V1 | Contra | 3.79 | 74 | 147 | 58 |  |
| V2/V3d | Ipsi | 4.15 | 126 | 155 | 58 |  |
| Cb | Ipsi | 2.5 | 107 | 143 | 51 |  |
| Cb | Ipsi | 3.313 | 108 | 148 | 24 |  |
| Cb/V2 | Contra | 3.52 | 89 | 165 | 57 |  |

The level of the coefficients was set at *P*<0.01 (t>2.38 for the intact stage, t>2.35 for the recovery stage). t-values at the center of individual masses of correlation (the locations are indicated with the positions along the x-, y- and z-axis) are indicated. See also Supplementary Figure 4.

**Supplementary Video Captions**

**Supplementary Video 1. Effect of the VSt lesion on finger dexterity (Monkey Sh).**

This video shows the performance of precision grip before and after the bilateral VSt lesion. The VSt lesion did not impede precision grip. This video was recorded before SCI.

**Supplementary Video 2. Effect of the temporary inactivation of the VSt on recovery time course of finger dexterity after SCI (Monkey R).**

Before SCI, the monkey grasped a morsel of sweet potato with precision grip. Immediately after SCI (postoperative day 1), the monkey could not retrieve a morsel. At postoperative day 16 after SCI, the monkey was able to grasp a morsel, though that grip was not a precision grip. At postoperative day 51 after SCI, precision grip recovered completely by daily rehabilitative training.

**Supplementary Video 3. Effect of the permanent lesion of the VSt on recovery time course of finger dexterity after SCI (Monkey Ju).**

Before SCI, the monkey grasped a morsel of sweet potato with precision grip. At postoperative day 7 after SCI, the monkey could reach a morsel. However, finger movements were not observed. At postoperative day 14 after SCI, the monkey grasped a morsel with a coarse grip. At postoperative day 27 and 56 after SCI, the monkey raked a morsel using the index finger, but thumb movements were not observed. This grip was categorized as coarse grip.

**Supplementary Video 4. Effect of the permanent lesion of the VSt on recovery time course of finger dexterity after SCI (Monkey Na).**

Before SCI, the monkey grasped a morsel of sweet potato with a precision grip. At postoperative day 2 after SCI, the monkey could reach a morsel. However, finger movements were not observed. At postoperative day 14 after SCI, the monkey raked a morsel by the index finger and grasped with the index finger and the nail of thumb. At postoperative day 56 after SCI, the monkey still raked a morsel using the index finger, but thumb movements were not observed. This grip was categorized as a coarse grip.

**Supplementary Video 5. Effect of the permanent lesion of the VSt on recovery time course of finger dexterity after SCI (Monkey Sh)**

This monkey showed the most severe impairment of finger movements after SCI. Before SCI, the monkey grasped a morsel of sweet potato with precision grip. At postoperative day 10 after SCI, the monkey could reach a morsel. However, finger movements were not observed. At postoperative day 24 after SCI, the monkey still could not grasp a morsel. At postoperative day 41 after SCI, the monkey raked a morsel by the index finger and dropped it, then grasped the dropped morsel with the index finger and the nail of thumb. This grip was categorized as a coarse grip.
